# Acute Effect of Transcutaneous Electroacupuncture on Globus Pharyngeus: A Randomized, Single-Blind, Crossover Trial

**DOI:** 10.3389/fmed.2020.00179

**Published:** 2020-05-12

**Authors:** Wencong Zhou, Qi Deng, Lin Jia, Hanbing Zhao, Meng Yang, Guoyuan Dou, Zijian He, Wanwei Guo

**Affiliations:** ^1^Department of Gastroenterology, Guangzhou First People's Hospital, Guangzhou Medical University, Guangzhou, China; ^2^Department of Gastroenterology, the Affiliated Hospital of Guizhou Medical University, Guiyang, China; ^3^Department of Gastroenterology, Guangdong Second Provincial General Hospital, Guangzhou, China

**Keywords:** traditional Chinese Medicine, globus pharyngeus, transcutaneous electroacupuncture, crossover study, meridian theory

## Abstract

**Background:** Acupuncture points are commonly used by Traditional Chinese Medicine to treat throat discomfort. Transcutaneous electroacupuncture (TEA) is a new therapy combining transcutaneous electrical nerve stimulation with meridian theory. The efficacy and mechanism of Transcutaneous electroacupuncture for globus pharyngeus has not been reported. The aim of our study was to explore the effect and possible mechanisms of TEA at CV22/LI3/LU11/ST36 for patients with globus.

**Methods:** A total of 80 patients with globus pharyngeus were randomly allocated into eight groups. The intervention order in Groups A1/B1/C1/D1 was firstly TEA at CV22/LI3/LU11/ST36 during the first period and sham-TEA in the second period. For participants in Groups A2/B2/C2/D2, the intervention order was the reverse. Before the test, the participants were asked to complete the Glasgow Edinburgh Throat Scale (GETS), visual analog scale (VAS), and the Hamilton Rating Scale Anxiety/Depression and were then asked to test and measure the heart rate variability and serum hormone levels of SP and NPY. At the end of the second period, these tests were manipulated again.

**Results:** D-values of GETS and VAS following stimulation at CV22/LU11 were significantly higher than those of sham-stimulating (CV22: 13.5 ± 13.09 vs. 1 ± 9.68, *P* <0.002; LU11: 17 ± 10.31 vs. 9 ± 9.68, *P* = 0.011). Heart rate variability, SP, and NPY were showed a significant difference in LU11 stimulation compared to other acupuncture points (*P* all <0.05).

**Conclusion:** Stimulation at CV22/LU11 significantly improved symptoms of globus. The results indicated that symptoms may be improved by stimulating the parasympathetic nervous system and secreting SP and NPY when stimulating at LU11. For CV22, it may improve symptoms by direct action on the throat. Stimulating at CV22/LU11 may be a potential therapy for treating globus.

## Introduction

Globus pharyngeus, presenting as a sensation of a lump or similar stuck in the throat, is regarded as defining esophageal disorders belonging to functional gastrointestinal disorders (FGIDs) and is ranked as A4 according to the Rome IV consensus criteria (1). The overall lifetime prevalence of globus was 21.5% (2), and it also has a tendency to recur. Its etiology, however, is still not clear, and psychological factors, abnormal upper esophageal sphincter (UES) function, smoking, sleep disorders, and gastrointestinal diseases have been suggested as potential causes of globus (2–4). Our previous research found that the low-dose amitriptyline or Paroxetine therapy was more efficacious than a proton-pump inhibitor for globus patients; the latter can be used for long-term management, but the antidepressants also had side effects, such as sleepiness, dizziness, and dry mouth (5–7). The patients with globus who failed to respond to a proton-pump inhibitor and antidepressants needed further treatment options.

Traditional Chinese Medicine (TCM) describes globus as “globus hystericus,” and has often acupunctured the Tiantu acupoint (CV22), Sanjian acupoint (LI3), Shaoshang acupoint (LU11), and Zusanli acupoint (ST36) and has also used a Banxia Houpu Decoction to relieve globus symptoms. Acupuncture is a therapeutic method that has been practiced for thousands of years in TCM's long history. It has been successful in treating various gastrointestinal diseases, especially FGID (8). In recent years, TEA, which uses surface electrodes instead of acupuncture needles, is a new therapy combining transcutaneous electrical nerve stimulation with meridian theory, and it has proven to be as effective as traditional acupuncture (9). The efficacy of TEA for gastrointestinal disorders has been reported by several studies, especially for Gastroesophageal reflux disease (GERD), functional dyspepsia (FD), irritable bowel syndrome (IBS), and Chronic Constipation, demonstrating a significant improvement in patients' symptoms as well as quality of life (10–12). However, little research has been conducted to investigate the effect of TEA on Globus pharyngeus, and the mechanism of this therapy is therefore, so far, not clear (13).

This study was a trial to explore the effect and possible mechanisms of TEA at CV22/LI3/LU11/ST36 for patients with globus through assessing the acute comprehensive effect of TEA, side effects, heart rate variability (HRV), and the plasma levels of certain gastrointestinal peptides.

## Materials and Methods

### Ethics Statement

This research—a prospective, randomized controlled trial for globus—conforms to the ethical guidelines of the 1975 Declaration of Helsinki (6th revision, 2008), and it was approved by the ethical review board of Guangzhou Nansha Central Hospital and registered at the Chinese Clinical Trial Registry center (Registration number: ChiCTR-IOR-16009196, Date of Registration: 12/09/2016). Written informed consent was obtained from all subjects before the research.

### Patients

We enrolled 80 patients with globus pharyngeus. All of them were out-patients who visited Division of Gastroenterology and Ear, Nose, and Throat of Guangzhou Nansha Central Hospital, Guangzhou First People's Hospital, Guangdong Second Provincial General Hospital, and the Affiliated Hospital of Guizhou Medical University from November 2016 to March 2018. All the patients satisfied the criteria: (1) meeting the Rome IV consensus criteria for the diagnosis of globus pharyngeus (1); (2) willingness to sign the informed consent form; and (3) were aged above 18 years. In addition, all patients underwent upper gastrointestinal endoscopy or laryngoscopy to exclude the presence of any organic disease.

However, patients were excluded if they fulfilled one or more items of the following list: (1) disagreed with the informed consent; (2) they exhibited esophagus, throat, nose, mouth, hypothyroid, brain, or organic heart organic disease identified by laboratory, upper gastrointestinal endoscopy, laryngoscopy, gastrointestinal barium meal, computed tomography, or electrocardiograph examination; (3) they wore a heart pacemaker; (4) they were pregnant; or (5) they had knowledge of acupuncture points.

### Grouping

The study was a single-blind randomized crossover trial. Eighty patients were randomly assigned to one of eight equal groups (group A_1_ ~ D_2_) by a computerized random number generator. The subjects were blinded to the acupuncture point that would be used in the following treatment, and their demographical data, previous medication use, and medical histories were collected before the treatment. Group A_1_/B_1_/C_1_/D_1_ were firstly treated with TEA at CV22/LI3/LU11/ST36 for 30 min, and this was followed by a 3-day wash out stage and treatment with sham-TEA at a sham-point for 30 min at the second stage. Group A_2_/B_2_/C_2_/D_2_ underwent treatment in a reverse order.

**Figure 1:**
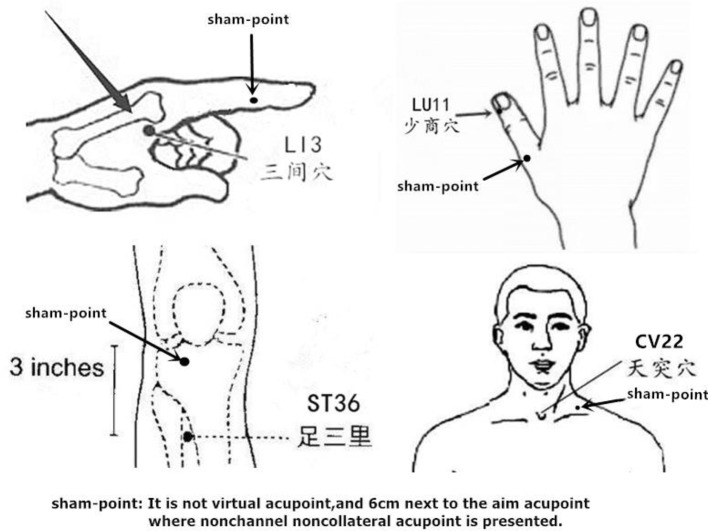
Location of points.

The watch-size microstimulator used to deliver TEA or sham-TEA was called Neuromodulation Regulator (SNM-FDC01, Ningbo Maida Medical Device, Inc., Ningbo, China). The electrical stimulus used for TEA or sham-TEA consisted of pulse trains with train on-time of 2 s and off-time of 3 s, pulse frequency of 25 Hz, pulse width of 0.6 ms, and amplitude 2–10 mA depending on the comfortability and tolerance of the patient. The aim acupoints are shown in Figure 1.

### Assessments

The GETS, VAS, and HRV were performed, and serum hormone levels of SP and NPY were measured by ELISA before the treatment was administered and at the end of the first and second stage. Moreover, side effects and alleviation time of symptoms were also assessed at the end of the first and second stages. The Hamilton Depression Rating Scale (HAMD) and the Hamilton Anxiety Rating Scale (HAMA) were used before the study to assess the baseline of the subjects' severity of depression and anxiety. The main efficacy endpoint included the GETS and VAS. The secondary efficacy endpoint included the HRV, HAMD, and HAMA.

1. The GETS (14) included 12 questions and was used to evaluate throat symptom severity. The scores of 12 questions comprise globus symptom scores and scores that assess the patient's psychological severity. In this study, we only calculated the globus symptom score, including scores of 10 questions on a seven-point Likert scale, with 0 being “none” and 7 being “unbearable.”
2. VAS (15) was extensively applied when evaluating the subjective feelings of patients. The scores of VAS ranged from 0 to 100, with 0 points indicating “absent” and 100 points indicating “extremely severe.”
3. HAMD (16), used in both clinical and research settings, was applied for evaluating the depression severity. The scale is composed by 17 items, and the total scores >7 points indicate the presence of depression.
4. HAMA (17), used in both clinical and research settings, was applied for assessing anxiety severity. The scale is composed of 14 items, and the total scores >7 points indicate the presence of anxiety.
5. HRV (18) is used to assess the autonomic function. A special one-channel amplifier (Ningbo Maida Medical Device Inc., Ningbo, China) amplifies the electrocardiogram (ECG) signal to the computer. The HRV signal was derived from the ECG. The figure of the low-frequency (LF) band is frequently employed as a measure of sympathetic tone and the figure of the high-frequency (HF) band represents vagal activities. The LF/HF ratio indicates the interaction of sympathetic nerve and vagal nerve and has a positive correlation with sympathetic nervous activity.
6. In the analysis of peptides: blood samples were collected and centrifuged at 3,000 g for 15 min. Plasma samples were collected and stored at −80°C. SP and NPY levels were measured by using commercial ELISA kits (Wuhan, Elabscience Biotech Co., Ltd., China).

### Processing

**Figure 2:**
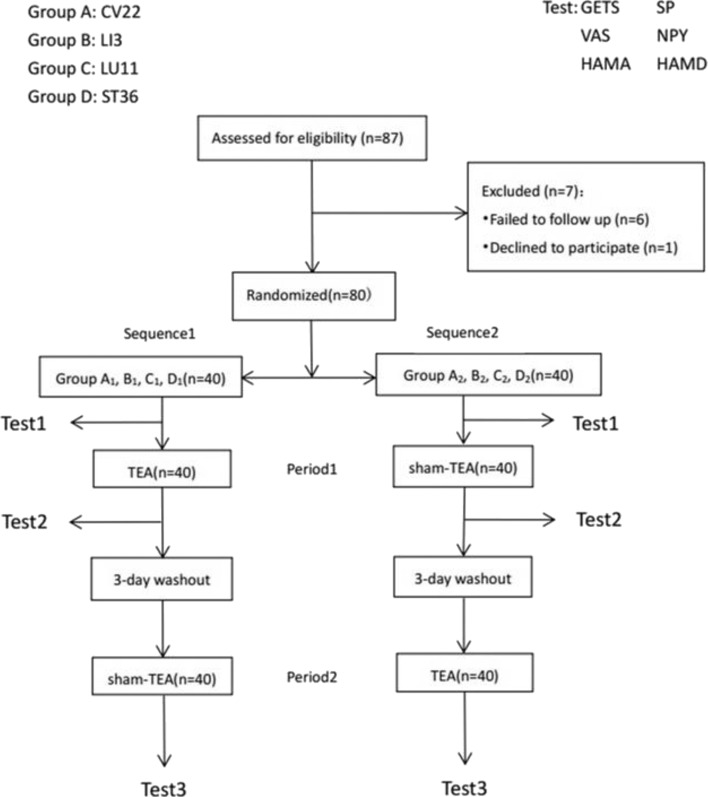
Flow-process diagram. Study design: each large group contained 20 patients that were equally and randomly divided into two smaller group. Treatment, sequences, and periods for each smaller group with a minimum 3-day washout period was set between administration period to minimize any TEA or sham-TEA carry-over effect. Because test 1 was the first test before any treatment, its results were also considered as the baseline of each sequence.

Before the test, participants were asked to complete GETS, VAS, HAMA, and HAMD and then to test the heart rate variability and serum hormone levels of SP and NPY, which were measured by ELISA. At the end of the second period, these tests were again to be manipulated. The intervention order in Groups A1/B1/C1/D1 was firstly TEA at CV22/LI3/LU11/ST36 for 30 min, followed with a 3-day wash out stage during the first period and sham-TEA at sham-point for 30 min in the second period. For participants in Groups A2/B2/C2/D2, the intervention order was reversed, with sham TEA in the first period and TEA in the second period. The target acupuncture was stimulated for 30 min, and heart rate variability was measured for 15 min (Figure 2).

### Sample Size

Previously (10, 19), a few studies have reported that the HF activity and plasma NPY of globus patients increased significantly after acute TEA, although patients in the group of each sequence were <10. For estimating the sample size of the study, a significance level of the test was set at 0.05, while the power of the test was 0.9,$N={\left[\frac{(t_{\frac{\alpha }{2}}+t_{\beta })S_{d}}{\delta }\right]}^{2}$. Twenty patients per group (total *n* = 80) were required. Additionally, drop-out rates in our previous study was 0%. Therefore, recruiting up to 20 patients for each acupuncture point in this study was deemed sufficient to enable us to recruit and randomize sufficiently.

### Statistical Methods

Data analysis was performed using SPSS 17.0 software (SPSS Inc., Chicago IL, United States), and continuous variables were presented as the mean ± SD. The ANOVA for crossover design was conducted for the two-period crossover design. Comparing the differences between before and after treatment used the paired-sample *t*-test and differences between TEA and sham-TEA used *t*-test. Count data were compared across groups using the χ^2^ test. All tests were two-tailed, and statistical significance was assigned for *P* <0.05.

## Results

### Study Participants

Recruitment and allocation of participants were summarized in Figure 2. Six patients were lost to follow up and one patient declined to participate. The dropping rate is 8.05%. A total of 80 patients with globus pharyngeus were enrolled in the study and randomized into Group A1,B1,C1, D1, A2, B2, C2, and D2. The baseline characteristics of the patients (*n* = 80) were shown in Table 1. There were no statistically significant differences among the groups in gender, age, symptom duration, scores of GETS, VAS, HAMD, and HAMA, and there were also no statistically significant differences among the groups in the ratio of HRV (LF/HF) or the levels of SP and NPY (*P* > 0.05) (Table 1).

**Table 1:** Demographic and baseline characteristics of study patients.

	**CV22**		**LI3**		**LU11**		**ST36**		**F**	***P***
	**TS**	**ST**	**TS**	**ST**	**TS**	**ST**	**TS**	**ST**		
Gender(Male/ Female)	5/5	4/6	3/7	3/7	5/5	4/6	4/6	3/7	0.271	0.963
Age (year)	47.6 ± 13.10	40.7 ± 16.12	45.8 ± 12.09	47.3 ± 14.48	40.9 ± 6.9	50.5 ± 16.85	43.7 ± 12.45	48.8 ± 16.06	0.678	0.69
Symptom duration(month)	18.9 ± 8.94	29.9 ± 23.32	30 ± 19.67	17.3 ± 4.08	17.5 ± 9.8	30.6 ± 22.74	37.8 ± 38.99	18.3 ± 10.01	1.549	0.165
GETS	12.8 ± 3.01	11.5 ± 3.44	14.2 ± 3.36	13.8 ± 2.74	14.5 ± 5.19	14.2 ± 4.49	13.3 ± 4.14	15 ± 4.64	0.787	0.601
VAS	46 ± 14.30	44 ± 17.76	54 ± 15.78	43 ± 19.47	43 ± 17.03	53 ± 12.52	48 ± 13.17	49 ± 13.70	0.760	0.623
HAMA	10.2 ± 6.03	8.1 ± 2.28	11.3 ± 4.27	10.1 ± 5.24	12.2 ± 5.05	11.5 ± 4.01	13.1 ± 5.72	11.4 ± 3.84	1.051	0.404
HAMD	5.5 ± 3.06	6.2 ± 3.65	9.8 ± 1.55	10.7 ± 2.91	10.8 ± 5.51	10.6 ± 5.80	10.6 ± 5.62	9.3 ± 5.68	2.251	0.070
HRV(LF/HF)	1.57 ± 1.15	2.07 ± 1.09	2.04 ± 1.27	1.97 ± 1.23	1.24 ± 0.82	2.74 ± 1.83	1.22 ± 0.82	1.47 ± 0.95	1.876	0.086
SP(pg/ml)	22.83 ± 17.51	28.38 ± 18.52	16.26 ± 8.99	19.60 ± 11.45	12.68 ± 3.38	46.89 ± 64.69	7.71 ± 3.16	23.75 ± 50.83	1.484	0.187
NPY(pg/ml)	2469.69 ± 1703.97	1816.21 ± 1790.15	2837.64 ± 1852.07	1768.09 ± 1720.15	2037.88 ± 1737.91	2837.64 ± 1852.07	2987.27 ± 1792.90	2381.78 ± 1784.70	0.724	0.652

*TS means sequence 1, TEA → sham-TEA; ST means sequence 2: sham-TEA → TEA*.

*Except gender, all the other data are expressed as $\bar{\text{X}}\pm \;\text{S}$*.

*There were no significant differences in all baseline characteristics of study patients*.

*GETS, Glasgow Edinburgh Throat Scale; VAS, Visual Analog Scale; HAMA, Hamilton Anxiety Rating Scale; HAMD, Hamilton Depression Scale; HRV, Heart Rate Variability; SP, Substance P; NPY, Neuropeptide Y*.

### Effects of TEA on the Symptoms of Globus Pharyngeus

Acute effect of TEA on CV22 and LU11 to alleviate the symptoms of globus pharyngeus

1. GETS: At LU11 and CV22, the results indicated that the scores of ΔGETS in TEA were significantly higher than those in sham-TEA, but they were not significant in ANOVA for the crossover design (*P*_LU11_ <0.001; *P*_CV22_ <0.001) (Table 3) or in the *t*-test (*P*_LU11_ <0.001; *P*_CV22_ <0.001) (Table 4). But the differences in the scores between the two periods and sequences were not statistically significant (LU11: *P*_1_ = 0.516, *P*_2_ = 0.517; CV22: *P*_3_ = 0.287, *P*_4_ = 0.588) (Table 3). ΔGESTS scores of TEA were 8.55 ± 3.20 (LU11) and 3.45 ± 1.85 (CV22), which were significantly higher than sham-TEA [3.8 ± 3.21 (LU11) and 0.8 ± 1.74 (CV22) (Table 2)]. Oppositely, there were no sequence, period and treatment effects on other acupuncture points (Table 3).
2. VAS: At LU11 and CV22, the results showed the scores of ΔVAS in TEA group were significantly different from those in sham-TEA group whether using the ANOVA for crossover design (*P*_LU11_ = 0.011; *P*_CV22_ <0.001) (Table 3) or *t*-test (*P*_LU11_=0.016; *P*_CV22_=0.002) (Table 4). The differences between the two periods and sequences were, however, not statistically significant (*P*_1_ = 0.729, *P*_2_ = 784; *P*_3_ = 0.402, *P*_4_ = 0.205). At LU11 and CV22, ΔVAS scores were significantly higher in TEA group than that in sham-TEA group (LU11: 17 ± 10.31 vs. 9 ± 9.68, *P* = 0.011; CV22: 13.5 ± 13.09 vs. 1 ± 9.68, *P* <0.002) (Table 2).
3. HAMA and HAMD: The ANOVA for the crossover design supported that the scores of ΔHAMA and ΔHAMD in TEA group had no significant differences in relation to the period, sequence, and treatment.

**Table 2:** Efficacy endpoint of the subjects after treatment.

**Index**	**Acupuncture point**	**Sequence**		**Period**		**Treatment**	
		**TS**	**ST**	**Period1**	**Period2**	**T**	**S**
ΔGETS	LI3	6.1 ± 2.86	6.7 ± 3.39	6.25 ± 3.52	6.55 ± 2.72	6.75 ± 3.39	6.05 ± 2.86
	LU11	6.6 ± 4.30	5.75 ± 3.69	6.4 ± 4.27	5.95 ± 3.75	8.55 ± 3.20	3.8 ± 3.21
	ST36	5.75 ± 3.75	1.55 ± 3.94	7.15 ± 4.36	6.15 ± 3.44	7.2 ± 3.61	6.1 ± 4.20
	CV22	1.95 ± 2.14	2.3 ± 2.34	1.85 ± 2.03	2.4 ± 2.42	3.45 ± 1.85	0.8 ± 1.74
ΔVAS	LI3	17 ± 17.20	12 ± 13.22	17 ± 13.80	12 ± 16.73	15 ± 13.57	14 ± 17.29
	LU11	13.5 ± 11.37	12.5 ± 10.20	13.5 ± 9.33	12.5 ± 12.09	17 ± 10.31	9 ± 9.68
	ST36	11 ± 8.52	16.5 ± 10.40	13.5 ± 8.75	14 ± 10.95	15 ± 10	12.5 ± 9.67
	CV22	4.5 ± 15.72	10 ± 9.18	8.5 ± 13.09	6 ± 13.14	13.5 ± 13.09	1 ± 9.68
ΔHAMA	LI3	3.95 ± 2.01	3.05 ± 1.19	3.3 ± 1.63	3.7 ± 1.78	3.75 ± 1.65	3.25 ± 1.74
	LU11	3.8 ± 1.54	4.25 ± 1.52	4.4 ± 1.67	3.65 ± 1.31	3.95 ± 1.36	4.1 ± 1.71
	ST36	6.75 ± 4.04	4.8 ± 2.48	5.7 ± 3.71	5.85 ± 3.27	5.9 ± 3.45	5.65 ± 3.55
	CV22	3.8 ± 3.69	1.95 ± 2.33	3.4 ± 3.36	2.35 ± 3.00	3.15 ± 3.39	2.6 ± 3.03
ΔHAMD	LI3	5.2 ± 1.54	5.25 ± 1.62	5.25 ± 1.65	5.2 ± 1.51	5.4 ± 1.43	5.05 ± 1.70
	LU11	5.85 ± 5.34	4.25 ± 4.58	4.5 ± 4.62	5.6 ± 5.37	5.25 ± 5.15	4.85 ± 4.92
	ST36	6.25 ± 4.40	3.8 ± 3.49	5 ± 4	5.05 ± 4.32	5.2 ± 3.75	4.85 ± 4.53
	CV22	1.95 ± 3.58	1.45 ± 4.61	2 ± 3.32	1.4 ± 4.78	2.15 ± 3.95	1.25 ± 4.25

*T, TEA; S, sham-TEA*.

*TS means sequence 1: TEA → sham-TEA; ST means sequence 2: sham-TEA → TEA*.

*All the data were expressed as $\bar{\text{X}}\pm \;\text{S}$*.

*GETS, Glasgow Edinburgh Throat Scale; VAS, Visual Analog Scale; HAMA, Hamilton Anxiety Scale; HAMD, Hamilton Depression Scale*.

**Table 3:** Sequence, period, and treatment effects on ΔGETS.

**Index**	**Acupuncture point**	**SS**	**Df**	**MS**	**F**	***P***
Sequence effect	LI3	3.60	1	3.60	0.207	0.655
	LU11	7.225	1	7.225	0.436	0.517
	ST36	32.40	1	32.40	1.604	0.222
	CV22	1.225	1	1.225	0.304	0.588
Period effect	LI3	0.90	1	0.90	0.293	0.595
	LU11	2.025	1	2.025	0.440	0.516
	ST36	10	1	10	1.018	0.326
	CV22	3.025	1	3.025	1.203	0.287
Treatment effect	LI3	4.90	1	4.90	1.598	0.222
	LU11	225.625	1	225.625	49.019	<0.001
	ST36	12.1	1	12.1	1.231	0.282
	CV22	70.225	1	70.225	27.935	<0.001

*ΔGETS, baseline GETS—after TEA/sham-TEA GETS*.

*In the Glasgow Edinburgh Throat Scale, treatment effect of LU11 and CV22 was significant (P <0.001). There were no sequence effect and period effect*.

**Table 4:** Differences between TEA and sham-TEA.

**Index**	**Acupuncture point**	**TEA**	**Sham-TEA**	***P***
ΔGETS	LI3	6.75 ± 3.39	6.05 ± 2.86	0.484
	LU11	8.55 ± 3.2	3.8 ± 3.21	<0.001
	ST36	7.2 ± 3.61	6.1 ± 4.2	0.38
	CV22	3.45 ± 1.85	0.8 ± 1.74	<0.001
ΔVAS	LI3	15 ± 13.57	14 ± 17.30	0.84
	LU11	17 ± 10.31	9 ± 9.68	0.016
	ST36	15 ± 10	12.5 ± 9.67	0.426
	CV22	13.5 ± 13.09	1 ± 9.68	0.002
ΔHAMA	LI3	3.75 ± 1.65	3.25 ± 1.74	0.358
	LU11	3.95 ± 1.36	4.1 ± 1.71	0.761
	ST36	5.9 ± 3.45	5.65 ± 3.54	0.822
	CV22	3.15 ± 3.39	2.6 ± 3.03	0.592
ΔHAMD	LI3	5.4 ± 1.43	5.05 ± 1.7	0.485
	LU11	5.25 ± 5.15	4.85 ± 4.92	0.803
	ST36	5.2 ± 3.75	4.85 ± 4.53	0.792
	CV22	2.15 ± 3.95	1.25 ± 4.25	0.492

*In the Glasgow Edinburgh Throat Scale, treatment effect of LU11 and CV22 was significant (P < 0.001)*.

*GETS, Glasgow Edinburgh Throat Scale; VAS, Visual Analog Scale; HAMA, Hamilton Anxiety Scale; HAMD, Hamilton Depression Scale*.

### Brain–Gut Peptides

**Figure 3:**
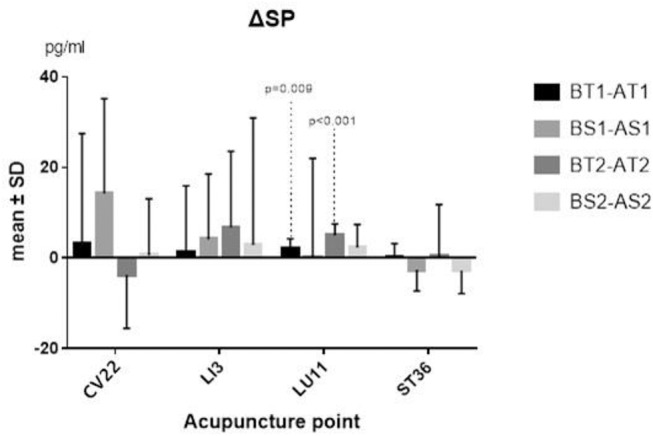
ΔSP. BT1 means before TEA in sequence 1; AS2 means after sham-TEA in sequence 2. Δ= baseline—after TEA/sham-TEA.

**Figure 4:**
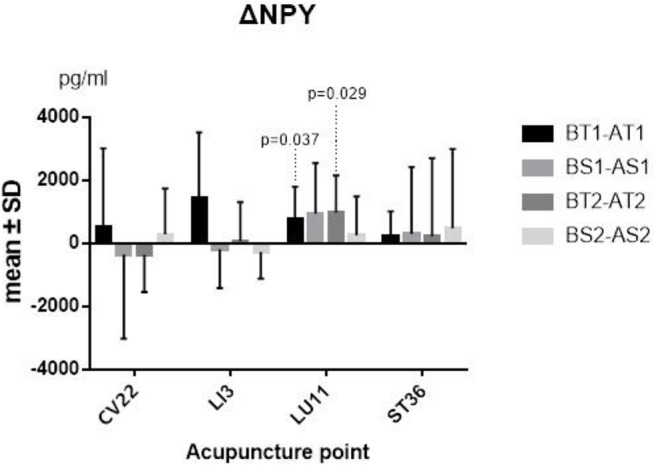
ΔNPY. BT1 means before TEA in sequence 1; AS2 means after sham-TEA in sequence 2. Δ= baseline—after TEA/sham-TEA.

The differences between before and after TEA of SP and NPY in serum of each group came from normal population, and a paired-sample *t*-test was thus adopted. The two sequences' average levels of ΔSP (*P* = 0.009; *P* <0.001) (Figure 3) and ΔNPY (*P* = 0.037; *P* = 0.029) (Figure 4) indicated that there were significant differences between before and after TEA when patients were stimulated at LU11, and the level of SP and NPY in the stage of after-TEA was higher than that of before-TEA at two tailed significant level 0.05. The four 95% confidence intervals of the difference [(0.716, 3.690), (3.121, 6.811), (56.748, 1504.091), and (124.10, 1814.87)] also supported that there was a significant difference between before and after TEA.

### Heart Rate Variability

**Figure 5:**
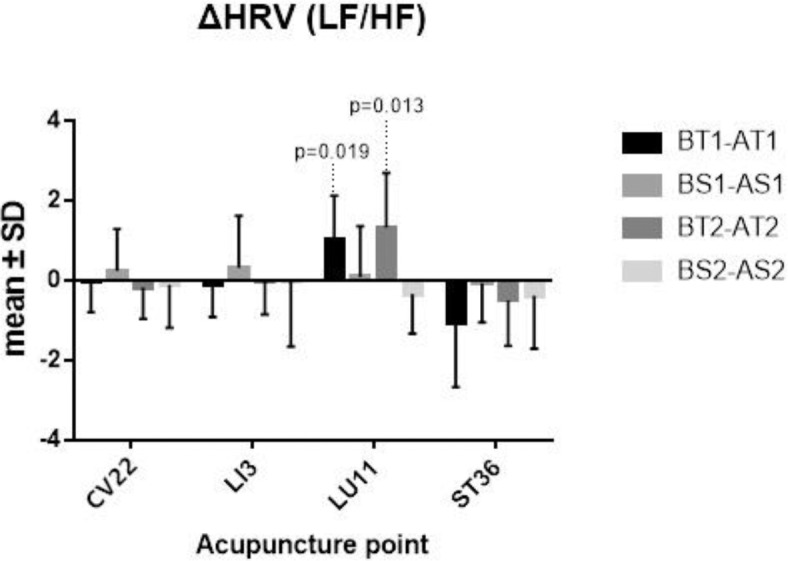
ΔHRV. BT1 means before TEA in sequence 1; AS2 means after sham-TEA in sequence 2. Δ= baseline—after TEA/sham-TEA.

Some differences of before and after TEA/Sham-TEA were chosen from the normal population at the significant level 0.10, and a paired-sample *t*-test was thus adopted. The others, which were not from the normal population, need to be tested, however, with a Wilcoxon matched-samples rank sum test. The results supported that there was a significant difference between before TEA and after TEA when patients were stimulated at LU11, either in Sequence 1 or Sequence 2 (*P* = 0.009; *P* <0.001) (Figure 5).

## Discussion

In this study, we found that the acute treatment with TEA at the acupoint of CV22 and LU11 significantly improved the globus pharyngeus patients' symptoms. Our previous studies demonstrated that only up to 35.7% of globus patients had a treatment response to a standard PPI dose once daily (6). The antidepressants represent the mainstay of treatment for globus pharyngeus (5, 6) since it is well believed that psychological disorders may be a significant factor in globus (20). But there were still 25% patients who failed to respond to antidepressants (6). In addition, the antidepressants also exhibited side effects, and some patients resisted the use of antidepressants because of them. As a result, further treatment options are needed for patients in the conditions described above.

In this study, we also found that patients with globus pharyngeus who were treated by TEA at CV22 and LU11 showed a significant decrease in scores of GETS and VAS compared with the corresponding sham-TEA. At the same time, the patients showed a good compliance of this therapy, and none of the patients quitted the study. Through searching the literature databases, this was the first study to investigate the efficacy of TEA for globus pharyngeus.

Some previous studies have reported the dysfunction of the autonomic nervous system (ANS) might play a role in the development of FD, IBS, or GRED (21–23) and also in the pathophysiology of psychological disorders involving anxiety or depression (24). HRV analysis is used as a non-invasive method of assessing autonomic activities (25). Liu et al. (10) observed that TEA could improve dyspepsia symptoms of FD patients, which is relevant to the increase of HF HRV and decrease of LF/HF HRV. Ouyang et al. (26) also reported that electroacupuncture significantly improved gastric emptying in association with the increase of HF HRV reflecting vagal activities. However, Huang et al. (27) did not find that TEA could influence sympathetic and/or parasympathetic nerve activity. Tatewaki et al. (28) showed that naloxone could inhibit the effects of TEA at ST36 on gastric accommodation in diabetic rats, suggesting another opioid mechanism of TEA. A few study results seem to support various mechanisms, such as the fact that the improvement of GI motility results from the activation of somatic and peripheral nerves by EA (29). This study also showed that TEA could alter the autonomic function as the LF/HF HRV changed before and after the treatment with LU11 but not other acupoints. We speculated that the reason for this might be that the particular methodologies of TEA are different, such as the use of different stimulation locations and parameters. Moreover, there might be various pathophysiological processes when TEA stimulates different locations, meaning different somatosensory neurons, which activate various nuclei at the central nervous system. Therefore, further research must be carried out to investigate the exact mechanisms behind the ameliorating effect of TEA on globus pharyngeus.

In recent years, the dysregulation of the brain–gut axis has been proven to be an important factor in FGIDs (1, 22). The brain–gut axis, where the psychosocial factors influence the gastrointestinal tract and vice versa, also exists in globus pharyngeus, as the severities of globus patients' symptoms and the mental disorders such as depression and anxiety have a positive correlation. In addition, a variety of brain–gut peptides and gastrointestinal hormones can influence the regulation of the axis. NPY and SP, known as brain–gut peptides, play a significant role in mental disorders of depression and anxiety in patients. Furthermore, therapies targeting NPY and SP might be a new way of alleviating the severities of stress and anxiety (30). Previous studies showed that TEA treatment could alter the level of NPY (10) and improved severities of stress and anxiety (19). In this study, we showed that the plasma NPY and SP levels were significantly altered by the TEA at LU11. The potential mechanism may be that NPY and SP can relieve the mental symptoms at the biological level, thereby alleviating the symptoms of pharyngeal sensation of blockage. At the same time, NPY can also relieve the contraction of the pharyngeal muscles by inhibiting muscle excitement. But stimulation at LI3, CV22, and ST36 did not trigger a change. The reasons might be, firstly, that the time of stimulation might be not have been enough to make the SP or NPY level changed to a threshold that could be measured, as the previous report (10) found the NPY levels changed after 2 weeks of TEA treatment. Secondly, TEA at LI3, CV22, and ST36 (31) might alter other brain–gut peptides but not NPY or SP. Studies consisting of longer trials and measuring other peptides that are able to find differences in brain–gut peptides may thus be required.

To be clear, though the mechanism of treatment with TEA at CV22 and LU11 for globus patients is currently not what it should be and is lacking a good explanation from the view point of modern medicine, and the autonomic nervous system dysfunction causes symptoms of globus still requires more in-depth research, it does not mean that the treatment is ineffective. The acupuncture at CV22 and LU11 have represented a true therapeutic effect in traditional Chinese medicine for a long time, and we also found that symptoms of patients with TEA at CV22 and LU11 significantly improved in this study. Because of the complicated pathogenesis of globus, multidisciplinary treatment, including TEA, traditional Chinese medicine, and antidepressants, may have a therapeutic potential for treating globus. However, there were also several limitations to our study. The major limitation of this study was the limited number of patients examined, leading to a component of selection bias. Furthermore, our study was not a double-blind study. In order to reduce these biases, GETS, VAS, HAMA, HAMD, and heart rate variability were performed by a single psychiatrist who was blinded to the allocation. Further investigations are needed to explore the exact mechanism of TEA for globus pharyngeus.

## Data Availability Statement

All datasets generated for this study are included in the article/supplementary material.

## Ethics Statement

The studies involving human participants were reviewed and approved by Guangzhou First Municipal People's Hospital, Guangzhou Nan Sha Center Hospital Affiliated to Guangzhou Medical university. The patients/participants provided their written informed consent to participate in this study.

## Author Contributions

LJ contributed an idea to this research. WZ and QD designed the study, analyzed data, wrote parts of the paper, and enrolled and treated patients. Several participants, including HZ, MY, GD, ZH, and WG enrolled and treated patients.

## Conflict of Interest

The authors declare that the research was conducted in the absence of any commercial or financial relationships that could be construed as a potential conflict of interest.
